# Implementation of machine learning into clinical breast MRI: Potential for objective and accurate decision-making in suspicious breast masses

**DOI:** 10.1371/journal.pone.0228446

**Published:** 2020-01-30

**Authors:** Stephan Ellmann, Evelyn Wenkel, Matthias Dietzel, Christian Bielowski, Sulaiman Vesal, Andreas Maier, Matthias Hammon, Rolf Janka, Peter A. Fasching, Matthias W. Beckmann, Rüdiger Schulz Wendtland, Michael Uder, Tobias Bäuerle

**Affiliations:** 1 Department of Radiology, Universitätsklinikum Erlangen, Friedrich-Alexander-Universität Erlangen-Nürnberg, Erlangen, Germany; 2 Pattern Recognition Lab, Department of Computer Science, Friedrich-Alexander-Universität Erlangen-Nürnberg, Erlangen, Germany; 3 Comprehensive Cancer Center Erlangen-EMW, Universitätsklinikum Erlangen, Friedrich-Alexander-Universität Erlangen-Nürnberg, Erlangen, Germany; Medical University of Vienna, AUSTRIA

## Abstract

We investigated whether the integration of machine learning (ML) into MRI interpretation can provide accurate decision rules for the management of suspicious breast masses. A total of 173 consecutive patients with suspicious breast masses upon complementary assessment (BI-RADS IV/V: n = 100/76) received standardized breast MRI prior to histological verification. MRI findings were independently assessed by two observers (R1/R2: 5 years of experience/no experience in breast MRI) using six (semi-)quantitative imaging parameters. Interobserver variability was studied by ICC (intraclass correlation coefficient). A polynomial kernel function support vector machine was trained to differentiate between benign and malignant lesions based on the six imaging parameters and patient age. Ten-fold cross-validation was applied to prevent overfitting. Overall diagnostic accuracy and decision rules (rule-out criteria) to accurately exclude malignancy were evaluated. Results were integrated into a web application and published online. Malignant lesions were present in 107 patients (60.8%). Imaging features showed excellent interobserver variability (ICC: 0.81–0.98) with variable diagnostic accuracy (AUC: 0.65–0.82). Overall performance of the ML algorithm was high (AUC = 90.1%; BI-RADS IV: AUC = 91.6%). The ML algorithm provided decision rules to accurately rule-out malignancy with a false negative rate <1% in 31.3% of the BI-RADS IV cases. Thus, integration of ML into MRI interpretation can provide objective and accurate decision rules for the management of suspicious breast masses, and could help to reduce the number of potentially unnecessary biopsies.

## Introduction

Breast cancer is the most frequent malignant neoplasm for women in the Western world [1]. Imaging plays a central role in the assessment of patients with suspected breast cancer, with the breast imaging reporting and documentation system (BI-RADS) being one of the most widely used documentation approaches worldwide [2]. For the BI-RADS IV category, the likelihood of malignancy ranges between 2% and 95% [2], and histological verification is required for final diagnosis. If the likelihood of malignancy exceeds 95%, the BI-RADS V category will be assigned, again with histological verification as management recommendation. This pragmatic approach helps to minimize the rate of missed cancers, but also results in a significant number of unnecessary biopsies in patients with benign lesions.

In order to reduce the number of unnecessary interventional procedures, additional imaging tests have been developed. One of the most promising modalities for this purpose is breast magnetic resonance imaging (MRI). Recent meta-analyses verified high diagnostic accuracy for MRI in the workup of non-calcified equivocal lesions [3] and suspicious microcalcifications [4] with the potential to safely rule out malignancy in these patients. Nevertheless, the use of MRI is not without controversy, as image interpretation is based on complex diagnostic information. Therefore, MRI is still regarded as an operator-dependent method, and considerable interobserver variability of the BI-RADS descriptors is well documented [5,6]. Moreover, BI-RADS is a formal lexicon and does not provide objective decision rules to integrate relevant information and provide a diagnosis [7,8]. Therefore, the final BI-RADS assessment rather represents a radiologist’s subjective rating.

Machine learning (ML) is a promising approach to solving this dilemma [9–11]. Research of ML in the field of MRI has verified its potential to detect complex interactions between lesion characteristics [9–11], allowing differentiation of lesions as either malignant or benign [10]. ML can assign scores to estimate the likelihood of malignancy and could thus provide decision criteria to rule out malignancy in suspicious breast lesions. We therefore investigated whether the integration of ML into MRI interpretation can provide objective and accurate decision rules for the management of suspicious breast masses, to ultimately help to reduce the number of unnecessary biopsies.

## Materials and methods

### Patients

This study complies with the Declaration of Helsinki. The Ethics Commission of the Friedrich-Alexander-Universität Erlangen-Nürnberg approved this study (request #314_17 Bc), and informed consent was waived because of the retrospective nature of the study.

Initially, we screened our institute’s database to identify patients who received breast MRI for further workup of suspicious or highly suspicious lesions upon complementary assessment. The following criteria were used:

MRI between 12/2013 and 06/2017 with BI-RADS IV or V rating after complementary assessment performed by two experts in breast imaging with >15 years of experience (n = 254). Assessment followed national guidelines and included mammography, ultrasound and clinical examination [12]. Patients who exhibited isolated non-mass enhancements (n = 35), lesions that were not histologically confirmed (n = 25) or lesions not detectable in MRI (n = 19) were excluded. Two patients were excluded due to an incomplete MRI protocol. Three patients featured bilateral findings, with one benign lesion on one side and a malignant finding on the contralateral side.

**Fig 1:**
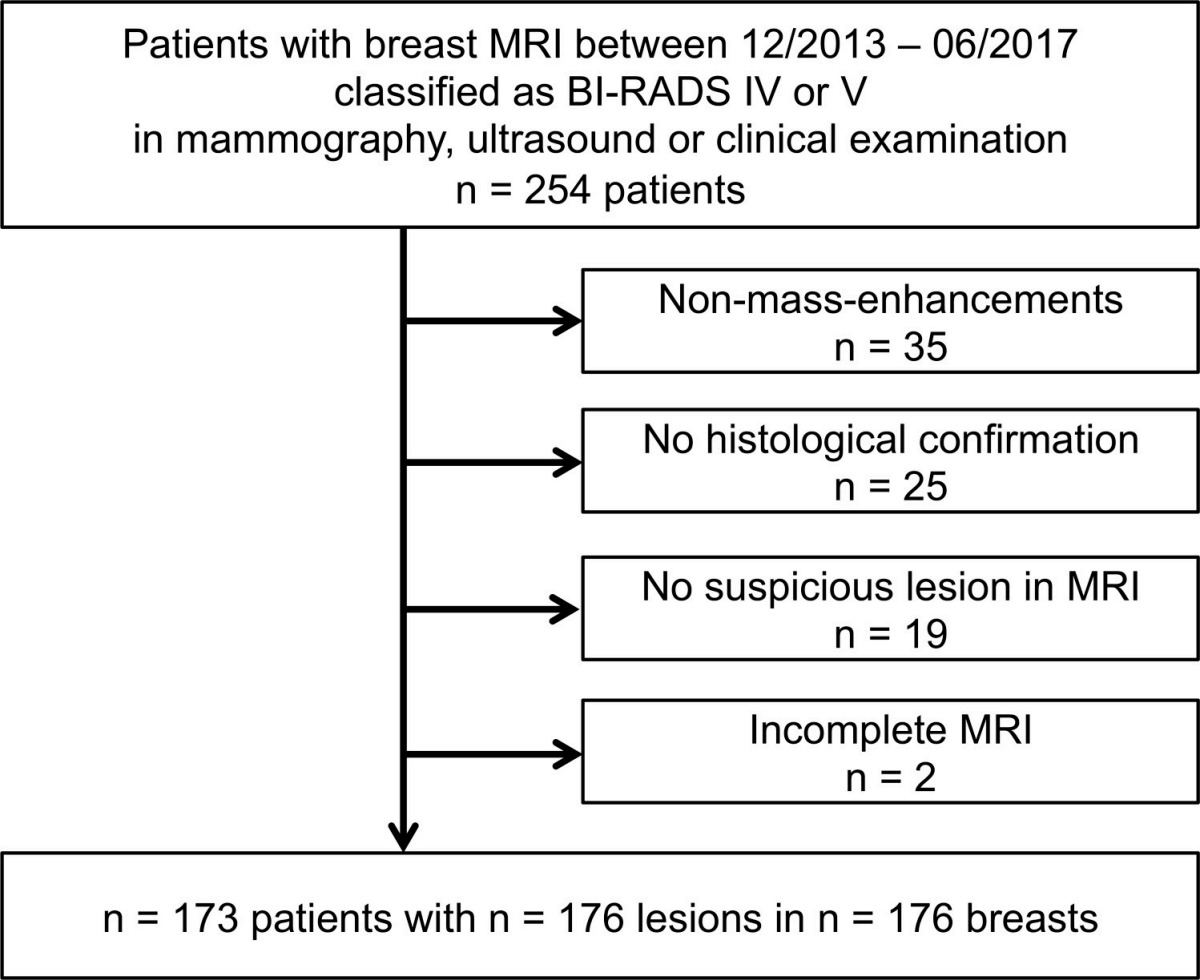
Patient flow chart. A database search revealed 254 patients having received breast MRI in our institute between 12/2013 and 06/2017 and classified as BI-RADS IV or V after complementary assessment by mammography, ultrasound or clinical examination. Exclusion criteria were: isolated non-mass-enhancements (n = 35), missing histological confirmation (n = 25), no suspicious lesion in MRI (n = 19), or an incomplete MRI protocol (n = 2). Applying the exclusion criteria resulted in n = 173 patients with n = 176 lesions in n = 176 breasts.

Thus, 173 patients with 176 suspicious or highly suspicious masses were included. Mean age was 54.3 ± 12.2 years (range: 26–85). Mean age of patients with malignancies was 58.1 ± 12.1 years (range: 32–85), and mean age of patients with benign lesions 48.5 ± 10.1 years (range: 26–73). A study population flowchart is presented in Fig 1.

### Standard of reference

All lesions were histologically verified by a board-certified breast pathologist. According to national guidelines, we used tissue samples from image-guided biopsy or surgical excision for histology [12]. Regarding the image-guided biopsies, sonography-guided 14-gauge core biopsy was performed in cases the lesion could be visualized by ultrasound. If the lesion could not be visualized by ultrasound, but in mammography, 9-gauge vacuum-assisted biopsy using stereotactic guidance was executed. All image-guided biopsies were performed by one of two board-certified breast radiologists, both with >15 years of experience (E.W. and R.S.-W.).

Pretest probability showed expected values [2]: 107/176 lesions (60.8%) were malignant, 69 lesions were benign (39.2%). Detailed pathological findings are provided in Table 1.

**Table 1:** Detailed pathological findings.

Benign (n = 69)			Malignant (n = 107)		
BI-RADS IV: n = 67; BI-RADS V: n = 2			BI-RADS IV: n = 33; BI-RADS V: n = 74		
Diagnosis	n^a^	%	Diagnosis	n	%
FMP	33	47.8%	NST	47	43.9%
FA	19	27.5%	NST+DCIS	33	30.8%
UDH	11	15.9%	DCIS	14	13.1%
SA	6	8.7%	ILC	13	12.1%
Papilloma	6	8.7%			
Lymph Node	3	4.3%			
Scar	2	2.9%			
Lobular Neoplasia	2	2.9%			
Others	4	5.8%			

FMP: fibrous mastopathy; FA: fibroadenoma; SA: sclerosing adenosis; UDH: usual ductal hyperplasia.

^a^Some benign lesions showed secondary histopathological diagnoses, e.g., in several cases, the pathology report described a fibroadenoma surrounded by fibrous mastopathy. Thus, the percentages sum up to >100%.

NST: no special type carcinoma; DCIS: ductal carcinoma in situ; ILC: invasive lobular carcinoma.

### MRI

The MRI protocol was optimized following international recommendations and current practice in breast MRI [5,13]. Images were acquired in axial plane and the patient in a prone position [14] using either 1.5  T or 3.0 T scanners (Magnetom Avanto/Aera; Verio/Skyra) and dedicated breast array coils (all hardware: Siemens Healthineers, Erlangen, Germany). Protocols included dynamic contrast-enhanced T1-weighted scans, a T2-weighted fat-saturated scan and diffusion-weighted imaging. Protocol parameters are provided in Table 2. The contrast media (0.1  mmol/kg body weight gadobutrol, Bayer Schering Pharma, Berlin, Germany) was injected into an antecubital vein after the first dynamic acquisition [14] with a flow of 2.0  mL/sec, followed by a 20 mL saline flush. After a 30-second delay, the remaining five dynamic acquisitions were scanned under identical conditions.

**Table 2:** MRI sequence parameters.

Field Strength	Sequence	FOV [mm^2^]	Resolution [mm^3^]	TR/TE/TI [ms]	Duration [min]
1.5 T	T2w STIR	340 × 340	0.8 × 0.8 × 4	4900/62/165	2.33
	Dynamic T1w GRE (6×)	360 × 360	0.8 × 0.8 × 1.5	7.7/4.77	1.07×6
	DWI SE-EPI SPAIR	340 × 170	1.8 × 1.8 × 4	5100/60/150	3.29
3.0 T	T2w STIR	340 × 340	0.8 × 0.8 × 4.0	3570/70/230	3.29
	Dynamic T1w GRE (6×)	360 × 360	0.8 × 0.8 × 1.5	5.97/2.46	1.03×6
	DWI SE-EPI SPAIR	350 × 185	1.8 × 1.8 × 2.5	4300/58	1.53

The protocol was optimized following international recommendations and current practice in breast MRI [5,13]. Images were acquired in axial plane. B-values were: 50, 400, and 800 s/mm^2^. FOV: Field of view. TR, TE, TI: Repetition, echo, inversion time. T: Tesla. STIR: Short Tau Inversion Recovery. GRE: Gradient echo. SE: Spin echo. EPI: Echo planar imaging. SPAIR: Spectral adiabatic inversion recovery.

### Imaging parameters

**Fig 2:**
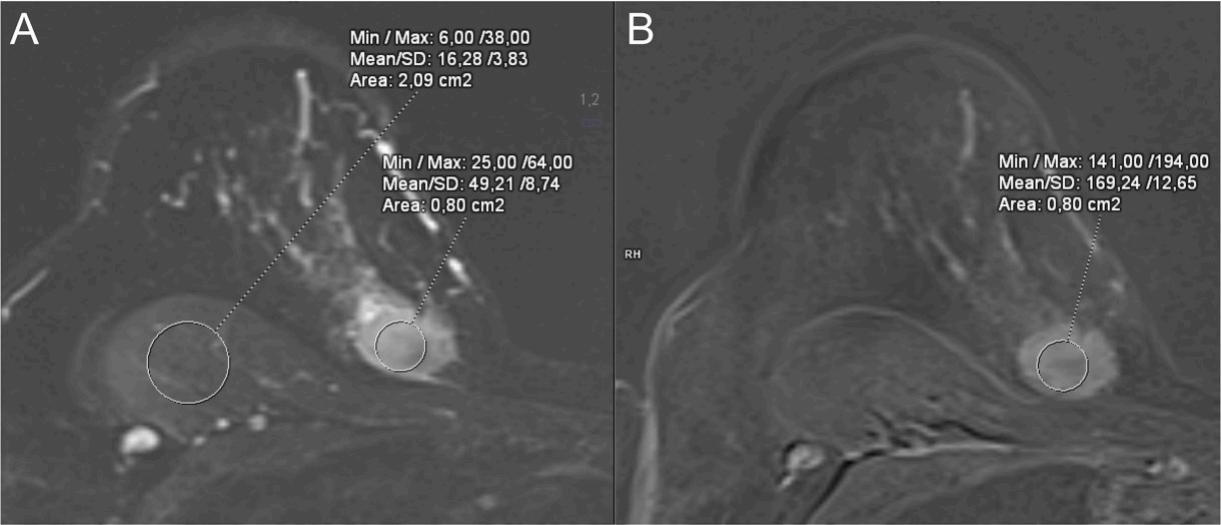
Assessment of T2w signal intensity (SI). (A) T2w SI was assessed on fat-saturated T2-weighted sequences. The ROI mask (see main task) was copied to this sequence from the contrast-enhanced T1w sequence (B). The corresponding mean SI was normalized to the mean SI of the pectoralis major muscle. In this example, this resulted in a T2w SI of 3.0 (49.2/16.3).

Breast MRI were assessed using a clinical post-processing platform (SynGo VIA V20A, Siemens Healthineers, Erlangen, Germany) by a radiologist with 5 years of experience in breast MRI (R1; S.E.) who was blinded to the standard of reference. Two board-certified breast radiologists with >15 years of experience (E.W. and R.-S.W.) supervised this process and ensured that the breast lesions on MRI were matched with the corresponding lesions on complementary assessment. R1 measured the lesion’s maximum diameter in axial orientation and the perpendicular diameter, and defined a circular region of interest (ROI) within the enhancing part of the lesion in the first post-contrast sequence, carefully avoiding the inclusion of non-enhancing lesion parts (e.g., cystic or necrotic compartments) and excluding surrounding tissue. Mean ROI size was 47.8 mm^2^ (range 4.7–103 mm^2^). This ROI served as a mask to be copied to the other sequences by the software. The following measurements were acquired to serve as potential predictors for malignancy:

- **Lesion size**: As lesion size correlates with likelihood of malignancy [15], we used the tumor’s dimensions as predictors: maximum diameter of the lesion [mm] in axial orientation (“long diameter”) and perpendicular diameter (“short diameter”).
- **Diffusion restriction**: The apparent diffusion coefficient (ADC) can be used to reduce the rate of unnecessary biopsies [16–18]. Mean ADC was measured as [10^−6^ mm^2^/s].
- **T2w signal intensity (SI)**: T2w SI, defined as a lesion’s signal intensity normalized to adjacent tissue such as the pectoralis major muscle, has been described as an adjunct feature to other BI-RADS diagnostic descriptors and has been shown to improve diagnosis in borderline BI-RADS categories [19,20]. As T2w SI can offer additional discrimination between malignant and benign lesions [19], we determined the lesion’s SI in the T2w fat-saturated sequence, followed by normalization to the SI of the pectoralis major muscle. For further explanation see Fig 2.
- **Lesion vascularity**:
  ○ Post-initial enhancement during the last versus first post-contrast scan was classified according to BI-RADS as type 1 (persistent increase: +10%), type 2 (plateau type: ± 10%), type 3 (washout: −10%) [2].
  ○ Contrast media washout is a biomarker of breast cancer [21] and was quantified as follows:

Washout rate = [1 − (SI_final_ / SI_max_)] × 100

SI was measured in the final scan and at the time point with maximum (max) enhancement during the dynamic series.

**Fig 3:**
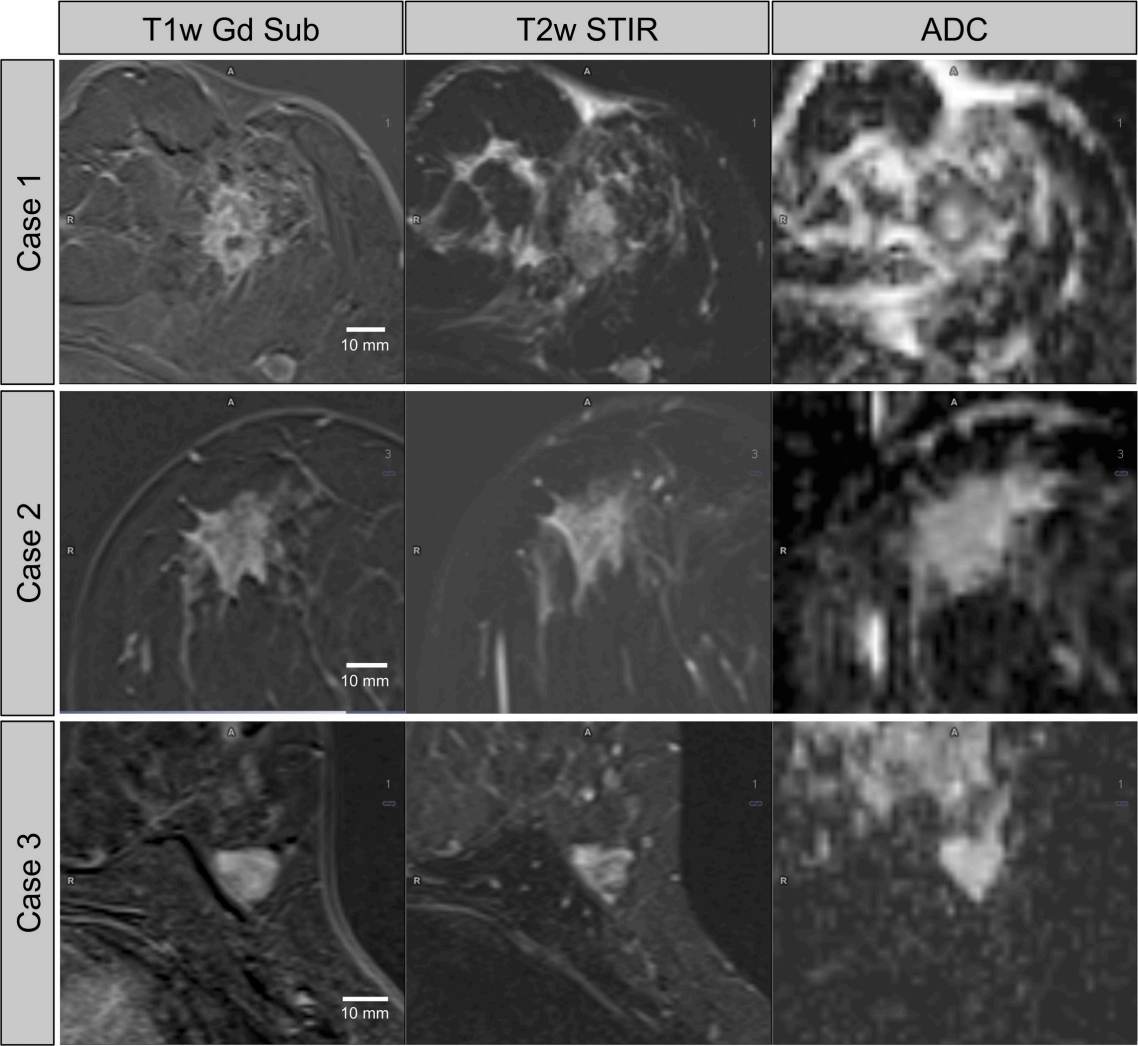
Clinical cases: Breast MRI of three different patients with suspicious lesions. Case 1: A 55-year-old woman presenting with a mass in her left breast, measuring 21 × 18 mm. ADC was 1015 × 10^−6^ mm^2^/s. There was a type-2 curve. T2w SI was 4.6. The SVM diagnosed malignancy (error rate / false positive rate: 2.9%). Histopathology: G3 NST. Case 2: A 50-year-old woman with a mass in her right breast, measuring 26 × 19 mm with an intermediate diffusion restriction (ADC 1300 × 10^−6^ mm^2^/s) and a type-2 curve. The T2w SI was 4.4. The SVM diagnosed malignancy (error rate / false positive rate: 15.9%). Histopathology: low-grade DCIS. Case 3: A 44-year-old woman with a mass in her right breast, measuring 15 × 15 mm without diffusion restriction (ADC 1545 × 10^−6^ mm^2^/s) and a type-1 contrast enhancement. The T2w SI was 12.7. The SVM excluded malignancy with an error rate / false negative rate of 2.8%. This diagnosis was correct and histopathology revealed a fibroadenoma.

All lesions were re-assessed by an inexperienced reader (R2, 5^th^-year medical student; C.B.) to determine interobserver variability. R2 was trained by R1 in lesion assessment on 20 sample cases not part of the study. Measurements from R2 were not used to train the algorithm. After assessment of all image parameters, the obtained parameters, the results from the pathology reports, and the BI-RADS classification from the radiology reports were combined to a comprehensive data table. Clinical cases demonstrating the acquired parameters and corresponding ML diagnoses are shown in Fig 3.

### Machine learning

An ML algorithm (polynomial kernel function support vector machine) was used for lesion classification [22]. These algorithms aim to define a decision boundary between two classes (e.g., benign vs. malignant lesion), based on input parameters. This decision boundary, also referred to as “hyperplane”, is orientated in such a way that it is as far as possible from the closest data points from each of the classes. These closest points are called support vectors [23]. Unlike other algorithms based on nonlinear optimization, the danger of getting trapped in local minima is low and the solution is unique and globally optimal [24,25]. The importance of the acquired features regarding classification was determined by calculating their information gain. The performance of the polynomial kernel function is influenced by hyperparameters–in particular, the polynomial degree and the cost variable. The latter controls the tradeoff between margin maximization and error minimization along with its scaling variable. ML optimization was focused on maximizing the area under the curve (AUC) of the Receiver Operating Characteristic (ROC). To determine the optimal hyperparameter combination for this task, a grid search was performed. To prevent overfitting and to ensure generalizability of the ML algorithm with regard to sample size and heterogeneity of the underlying biology, a ten-fold cross-validation approach was chosen. The ML algorithm was programmed in RStudio 3.4.1 [26] by S.E., M.D., S.V., and A.M., using the caret package [22]. Cross-validation was performed using the respective built-in function.

### Statistical analysis

Statistical analyses were performed using RStudio 3.4.1 [26]. Mann-Whitney U and chi-square tests were applied for intergroup comparisons of continuous and categorical variables, respectively. ROC curves were compared using DeLong tests. Interobserver agreement was determined by the intraclass correlation coefficient (ICC), with ICC>0.75 rated as “excellent” [27]. To estimate a systematic bias between the two readers, Bland-Altman plots [28] were generated by graphing the difference of each obtained parameter on the vertical against the absolute measurements of the two readers on the horizontal. Correlations were assessed using Pearson tests. In all statistical tests, p values <0.05 were considered significant. Confidence intervals (CI) were calculated at a confidence level of 95%.

ROC analysis was performed for all acquired parameters and the ML algorithm. ROC-AUC was used to estimate diagnostic accuracy. The ML algorithm’s performance was further analyzed by contingency tables including standard parameters of diagnostic accuracy and 95% confidence intervals (CI). In a similar fashion, a subgroup analysis for BI-RADS IV lesions was conducted.

In addition, potential decision rules were evaluated. Such decision rules have been described as promising tools for breast MRI assessment [7,29], enabling exact diagnostic statements of either presence (rule-in) or absence of malignancy (rule-out). As a common principle, if sensitivity is high, a “negative” test result will rule out malignancy, and with a high specificity, a “positive” test result will rule in malignancy [30–32]. Thus, rule-in and rule-out criteria were defined as follows:

**Rule-out criteria** were present if the SVM excluded breast cancer with an error rate <1%. Hereby, “error rate” is defined as the false negative rate (FNR = number of false negatives / standard of reference positives = 1 − sensitivity) [%]. In addition, rule-out criteria were determined for error rates <2%, <3%, <4% and <5%.

Likewise, **rule-in criteria** were explored at an error rate <1%. In this case, “error rate” was defined as the false positive rate (FPR = number of false positives / standard of reference negatives = 1 − specificity) [%]. Similarly, rule-out criteria were determined for error rates <2%, <3%, <4% and <5%.

### Open-access internet application

The ML algorithm was implemented into an open-access internet application with Shiny [33] to allow easy verification of our results on other cases. For any given lesion, this application provides a diagnosis (benign or malignant) based on the provided parameters. The diagnostic accuracy is further specified by the corresponding “error rate”. The results are moreover graphically visualized with the lesion’s coordinates highlighted on the ROC curve.

## Results

### Acquired parameters

**Fig 4:**
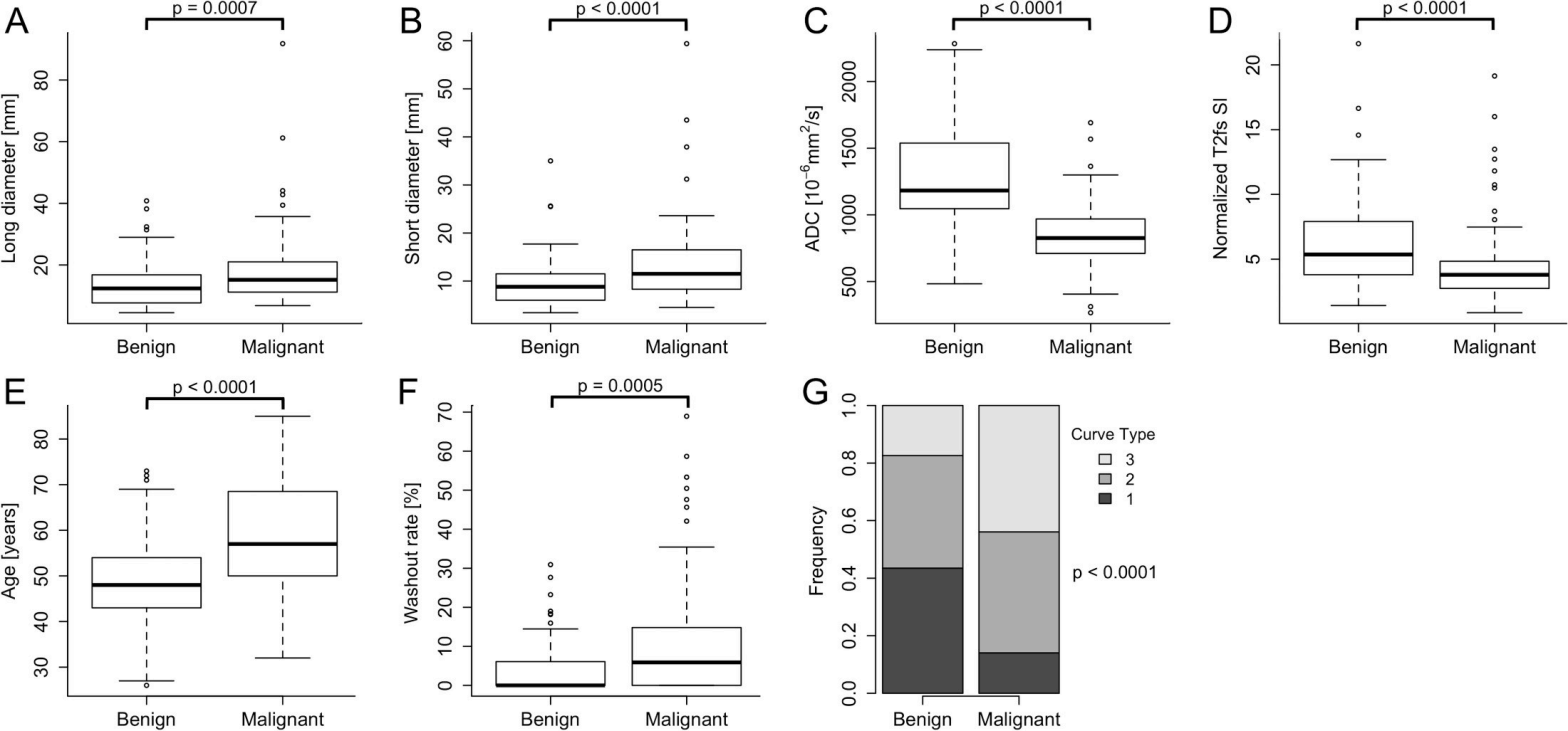
Acquired parameters in benign and malignant lesions. As demonstrated by boxplots (A to F) and the stacked column chart (G), all parameters showed significant potential for differential diagnosis (all, p ≤ 0.0007).

Malignant lesions featured higher long and short diameters, lower ADC values and lower T2w SI (all, p < 0.0001). Washout rate was higher in malignant lesions (p = 0.0005). The most frequent curve type of benign lesions was type 1 (43.5%, malignant: 14.0%; p < 0.0001), while type-3 curves were typical for cancers (43.9% vs. 17.4%; p < 0.0001). Patients with malignant lesions were significantly older compared to patients with benign lesions (median age 57 vs. 48 years; p < 0.0001). The acquired parameters showed excellent interobserver variability (ICC: 0.81–0.98) with variable diagnostic accuracy (AUC: 0.65–0.82). For details, compare Fig 4 and Table 3. A Bland-Altman analysis of the parameters is provided in S1 Fig.

**Table 3:** Diagnostic performance of the acquired parameters.

Parameter	Median *(IQR)*		ROC *(CI)*	Optimal cutoff	Sensitivity/ Specificity	ICC
	Benign	Malignant				
Patient age [years]	48 *(43–54)*	57 *(50–68*.*5)*	0.73 *(0*.*65–0*.*80)*	56.5	50% / 86%	n.a.
ADC [10^−6^ mm^2^/s]	1183 *(1046–1538)*	826 *(711–969*.*5)*	0.82 *(0*.*75–0*.*88)*	1038.5	85% / 75%	0.96
Long diameter [mm]	12.4 *(7*.*7–16*.*8)*	15.2 *(11*.*2–21*.*0)*	0.65 *(0*.*57–0*.*74)*	8.2	94% / 33%	0.98
Short diameter [mm]	8.8 *(6*.*0–11*.*5)*	11.5 *(8*.*3–16*.*5)*	0.69 *(0*.*61–0*.*77)*	11.3	54% / 74%	0.96
Curve type	n.a.		0.70 *(0*.*62–0*.*77)*	1 vs. (2 + 3)	86% / 43%	0.90
Washout rate [%]	0 *(0*.*00–6*.*07)*	5.88 *(0*.*00–14*.*80)*	0.65 *(0*.*57–0*.*73)*	1.4	67% / 61%	0.81
T2w signal intensity	5.4 *(3*.*8–7*.*9)*	3.8 *(2*.*7–4*.*8)*	0.68 *(0*.*60–0*.*76)*	5.1	79% / 58%	0.84

**Note:** All acquired parameters significantly differentiated benign and malignant lesions (all, p ≤ 0.0007). **IQR:** Interquartile Range. **ROC:** Receiver Operating Characteristic. **CI:** 95% confidence interval. For any given parameter, an optimal cutoff value was calculated from the ROC curve (maximizing the sum of sensitivity and specificity), with the particular sensitivities and specificities for the provided cutoffs given in addition. **ICC**: Intraclass correlation coefficient to estimate the interreader-agreement between reader 1 and reader 2.

### Machine learning

Feature selection identified all acquired parameters as significant predictors for the ML algorithm. The hyperparameter grid search for the SVM returned an optimal polynomial degree of 2. The cost variable was determined to be optimal at 0.39, with a scaling variable of 0.14.

**Fig 5:**
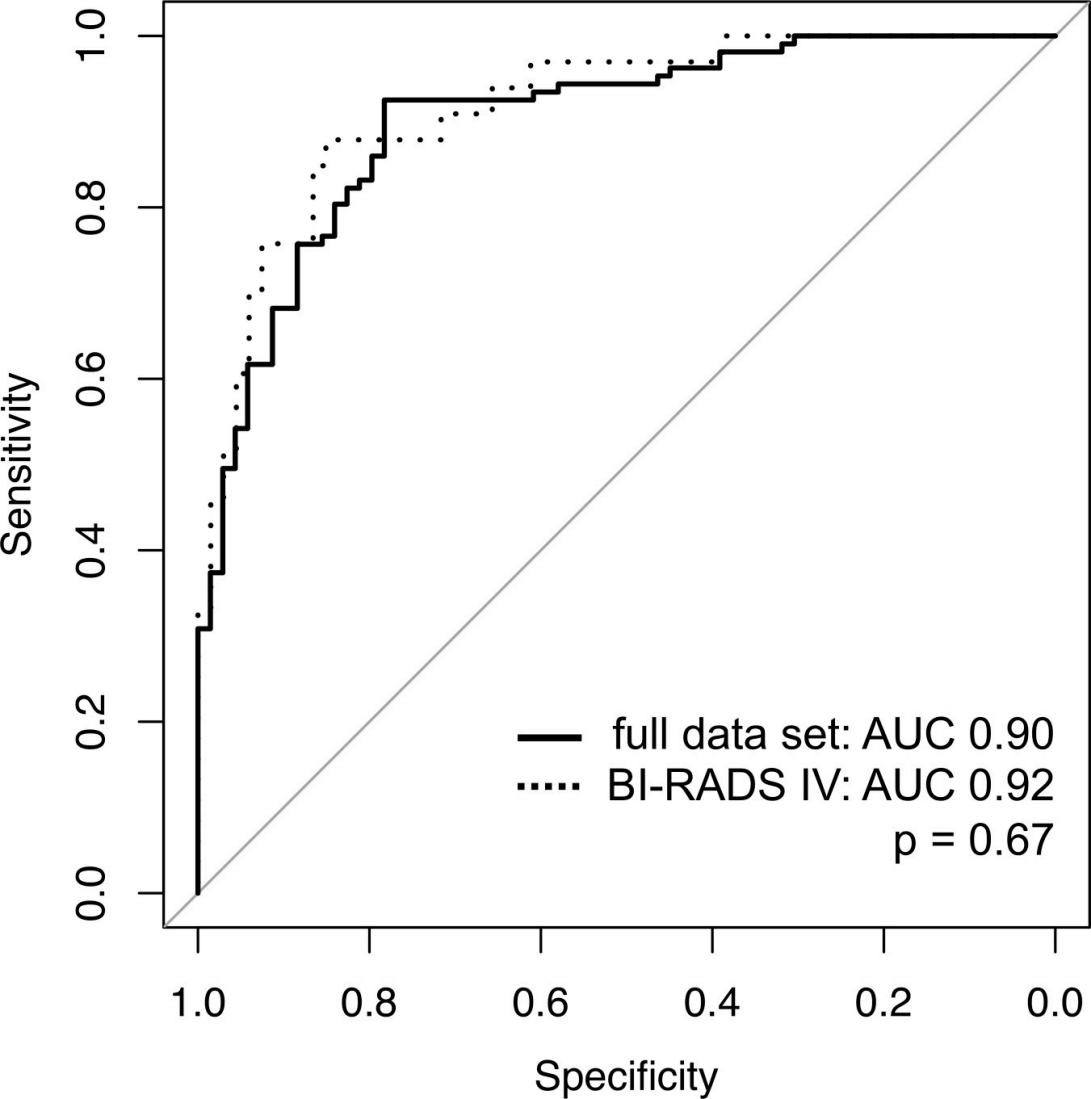
ROC analysis of the Machine Learning Algorithm. The diagnostic performance in the entire study collective (full data set: AUC = 0.90; black line) and the subset of BI-RADS IV cases (AUC = 0.92; black dotted line) was very good, without significant difference (p = 0.67).

Ten-fold cross-validation identified an accuracy of AUC = 90.1% for the ML algorithm (CI: 85.5–94.6%). Corresponding values of sensitivity (92.5%), specificity (76.8%), and positive and negative predictive value (PPV/NPV; 86.1% and 86.9%, respectively) confirmed the potential to differentiate between benign and malignant lesions. The ML algorithm significantly outperformed all individual semantic parameters (all, p ≤ 0.05). For detailed accuracy measures, see Table 3 and Fig 5.

**Fig 6:**
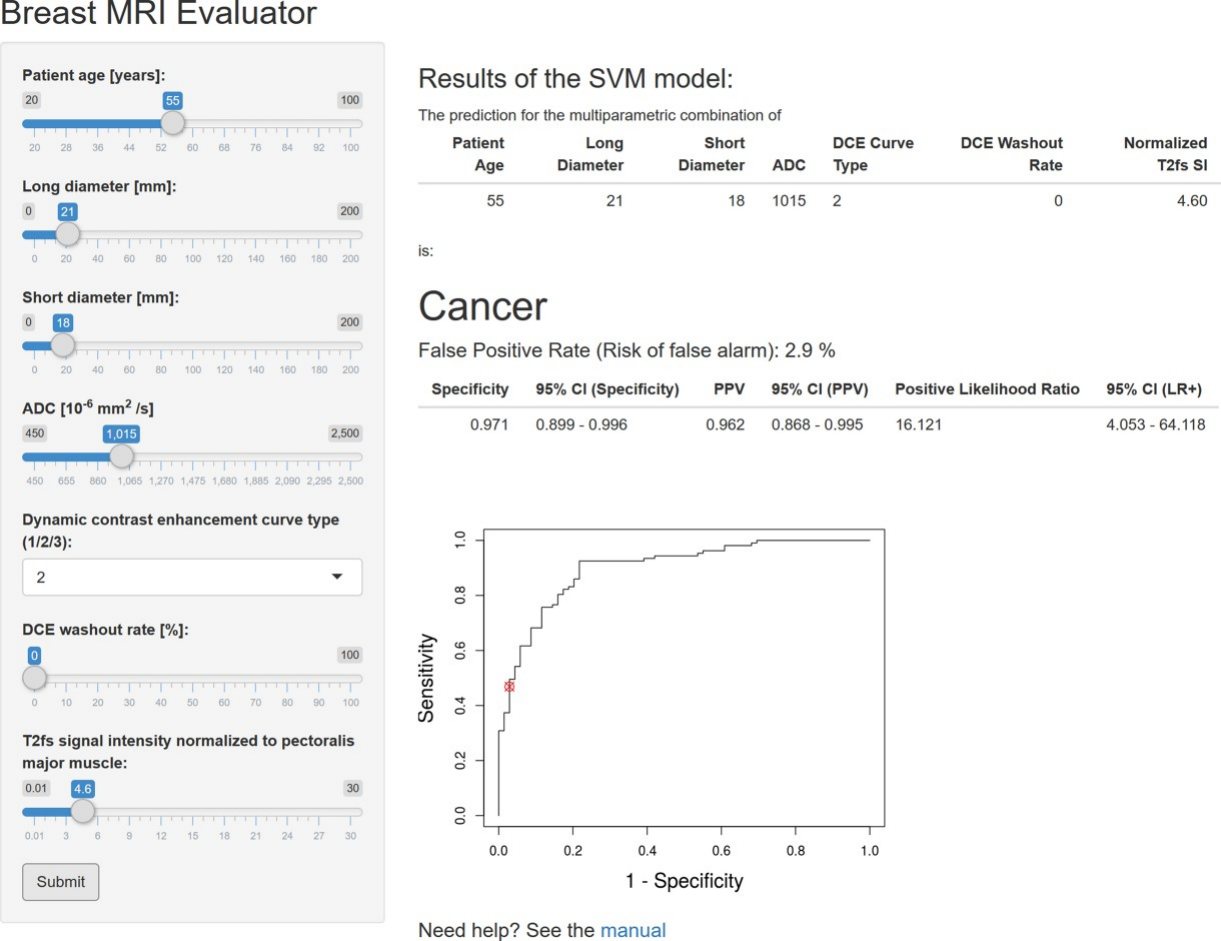
Exemplary output of the open-access internet application. This application can be used to verify our results and to translate our findings into a clinical setting. It can be accessed at: http://bit.do/Breast-MRI In this screenshot, the results of case #1 of Fig 3 are demonstrated. The Machine Learning algorithm predicted malignancy with an error rate (false positive rate) of 2.9%, a PPV of 96.2% and a specificity of 97.1%. Histopathology revealed a G3 NST carcinoma.

Rule-in criteria with an FPR <1% showed a prevalence of 33.6% (36/107). Rule-out criteria providing an FNR <1% were present in 30.4% (21/69; Table 4). The ML algorithm was implemented in an open-access internet application (Fig 6) and can be accessed at http://bit.do/Breast-MRI.

**Table 4:** Rule-in and rule-out criteria provided by the machine learning algorithm.

Error rate	Patients			
	Rule-in		Rule-out	
	All	BI-RADS IV	All	BI-RADS IV
<5%	58/107 (54.2%)	15/33 (45.5%)	35/69 (50.7%)	35/67 (52.2%)
<4%	53/107 (49.5%)	13/33 (39.4%)	34/69 (49.3%)	34/67 (50.7%)
<3%	53/107 (49.5%)	13/33 (39.4%)	24/69 (34.8%)	24/67 (35.8%)
<2%	42/107 (39.3%)	11/33 (33.3%)	24/69 (34.8%)	24/67 (35.8%)
<1%	36/107 (33.6%)	8/33 (24.2%)	21/69 (30.4%)	21/67 (31.3%)

The ML algorithm created decision rules to predict breast cancer (rule-in criteria). The diagnostic radiologist can choose the maximum accepted error rate (<1% to <5%). Decision rules apply only to a subset of the lesions, and generalizability decreases with decreasing error rates. For instance, 33.6% of all breast cancers (36/107) could be accurately diagnosed as malignant (error rate <1%).

The ML algorithm created additional decision rules to rule out the presence of breast cancer (rule-out criteria). Again, the diagnostic radiologist can choose the maximum accepted error rate (<1% to <5%). For instance, 31.3% of the BI-RADS IV ratings upon complementary breast assessment could be accurately diagnosed as benign, with an error rate <1% (21/67). Rule-out criteria could be used to reduce the rate of unnecessary biopsies in suspicious lesions.

### Subgroup analysis of BI-RADS IV lesions

A total of 56.8% of the lesions (100/176) were classified as BI-RADS IV (n = 33 malignant; n = 67 benign). Ten-fold cross-validation identified an accuracy of 91.6% for the ML algorithm (CI: 85.0–96.8%). The ML algorithm performed equally accurate in this subgroup compared to the entire study collective (p = 0.67; Fig 5). Corresponding values of sensitivity (87.9%), specificity (79.1%), and PPV/NPV (93% and 82%, respectively) again verified the potential to differentiate between benign and malignant BI-RADS IV lesions. Within the BI-RADS IV subgroup, rule-in criteria with an FPR <1% applied to 24.2% (8/33). Rule-out criteria providing an FNR <1% were present in 31.3% (21/67; Table 4).

### Interobserver agreement

The excellent interobserver variability between R1 and R2 regarding the acquired predictor parameters (compare Table 3) resulted in an excellent agreement with respect to the final diagnoses made by the SVM (ICC: 0.926; CI: 0.902–0.945). Moreover, the probability score output of the SVM strongly correlated between R1 and R2 (r = 0.943; p = 9.0 × 10^−85^; S2A Fig), which in turn resulted in no significant difference between the respective ROC curves (p = 0.17; S2B Fig).

## Discussion

Integration of ML into MRI interpretation provided objective and accurate decision rules for the management of suspicious/highly suspicious breast lesions. The presented ML algorithm achieved high diagnostic accuracy, particularly in BI-RADS IV findings. This result is of clinical importance, as the algorithm was based on a manageable amount of image features that could be reliably determined even by an inexperienced reader, as demonstrated by excellent ICC. It should be noted that this inexperienced reader was not a radiologist, but a medical student without previous experience in diagnostic imaging. Breast MRI is, however, usually regarded as a highly observer-dependent method, with high diagnostic performance most frequently reported in expert reading studies [3–5]. The excellent interobserver variability regarding the parameter acquisition translated into an excellent agreement between R1 and R2 regarding the final diagnoses established by the SVM. These results further underline the easy application of the ML algorithm in the assessment of suspicious breast lesions.

An additional important advantage of the presented ML algorithm is the implementation of decision rules. Such rules might help to solve the diagnostic dilemma that especially in the context of BI-RADS IV lesions with their likelihood of malignancy ranging between 2–95% [2], histological workup is typically recommended to reliably rule out breast cancer. Pathology reports however return benign findings in a significant number of patients. This situation is not satisfactory, and use of breast MRI has been suggested to reduce the number of unnecessary interventional procedures in these patients [3,4,34], with a recent meta-analysis reporting a sensitivity of 99% and an NPV of 100% for those lesions [3]. Nevertheless, most of the pooled studies were conducted by expert readers and – besides the mere absence of enhancement–there are no generally accepted criteria that define a “negative breast MRI” [3,4,34].

ML algorithms can be successfully applied to breast MRI [9,10] and offer the possibility of generating probabilistic results. The decision rules presented in this study allow the reader to either accurately diagnose (rule in) or exclude (rule out) malignancy at flexible thresholds in terms of the desired error rates [8]. As is to be expected, the decision rules were not applicable to all patients. Nevertheless, they could be applied for up to 54.2% of our patients. Most notably, rule-out criteria performed best in the subgroup of BI-RADS IV cases: In this subgroup, 31.3% of the benign lesions could be diagnosed with an FNR <1%. Hence, ML could assist the reader in making objective and accurate decisions in BI-RADS IV cases and thereby reduce the number of unnecessary biopsies by up to 31.3%.

The literature reports a number of classification rules for breast MRI [7,16,29,35–37], with most of them applying standard morphological criteria, and establishing a diagnosis by e.g. calculating simple sum scores [36,37]. ML, however, is able to detect interactions of higher complexity between imaging parameters, which translates into high accuracy [9,10]. Another approach different from sum score-based methods is the “Kaiser Score”, which represents one of the best investigated classification algorithms in breast MRI [7,29,35]. In contrast to our approach, it aims for a quick visual assessment that omits any measurements, is based on a decision tree algorithm and has been validated in multiple centers. Studies verified its low interobserver variability and high diagnostic accuracy [7,29,35].

Of note, our study did not apply any morphologic criteria for lesion analysis. Though it has been shown that morphologic parameters are key to an accurate diagnosis in breast MRI [7,29,35–37], a limitation of these descriptors is their potentially high observer-related bias [6]. This effect was considerably reduced in our study by using quantitative and semi-quantitative parameters, featuring interobserver variabilities well below the values reported in the literature [6]. Another approach and promising technique to exclude observer-related bias would have been the use of fully automated image analysis, which has been proven to be feasible in breast MRI [38,39]. Nevertheless, these deep-learning-based techniques are still under development and will probably not be freely available in the near future [11]. Therefore, our strategy was to include easily extractable parameters available with standard picture archiving and communication systems, and create an online accessible ML algorithm, thus rendering additional soft- or hardware unnecessary. This approach facilitates the translation of our results towards a clinical application.

Our current results are however limited due to the patient selection criteria: We investigated only BI-RADS IV and V lesions. Accordingly, the algorithm cannot be used in BI-RADS III and needs to be further validated for those lesions in upcoming studies. Non-mass lesions were not evaluated in the present analysis. These lesions are usually more difficult to differentiate compared to mass lesions [40,41]. We believe our ML algorithm might also be helpful in the evaluation of non-mass lesions and we are currently investigating this hypothesis. Non-enhancing lesions were excluded from the analysis. It has been proven that the absence of significant enhancement in MRI almost certainly excludes breast cancer [4,7]. Accordingly, the exclusion of 19 non-enhancing lesions likely decreased the actual diagnostic performance in our study group.

In conclusion, integration of ML into MRI interpretation provided objective and accurate decision rules for the management of suspicious and highly suspicious breast masses. In lesions rated as BI-RADS IV upon complementary breast assessment, this bears the potential for safely reducing biopsy rates by almost one third. The developed ML algorithm was made publicly available as an internet application and its results can be easily translated into clinical practice. This allows further prospective validation, which should be performed in future studies.

## Supporting information

S1 Fig Bland-Altman plots of the image parameters. Systematic biases of the image parameter assessments between Reader 1 and 2. Bland-Altman plots depicting differences of the parameters against the average measurements, with mean difference (purple line) and 95% limits of agreement (red lines). The regression lines (in dark green) proved not to be significant for all parameters (-0,81 ≤ all slopes ≤ 0.03; all, p ≥ 0.222).
(TIFF)

S2 Fig Diagnoses made by the Machine Learning algorithm—Comparison between Reader 1 and 2. (A) Pearson plot depicting the correlation between the probability score outputs of the SVM algorithm for both readers (R1 and R2, respectively). Probability scores correlated strongly and highly significantly (r = 0.943; p = 9.0 × 10^−85^). (B) Receiver Operating Characteristic plots for R1 (black) and R2 (gray) with no significant difference (p = 0.17).
(TIFF)
