# Supplementary figures and images for: Implementation of machine learning into clinical breast MRI: Potential for objective and accurate decision-making in suspicious breast masses
Source: PLoS One. 2020 Jan 30;15(1):e0228446. doi: 10.1371/journal.pone.0228446 (PMC6992224; doi:10.1371/journal.pone.0228446)

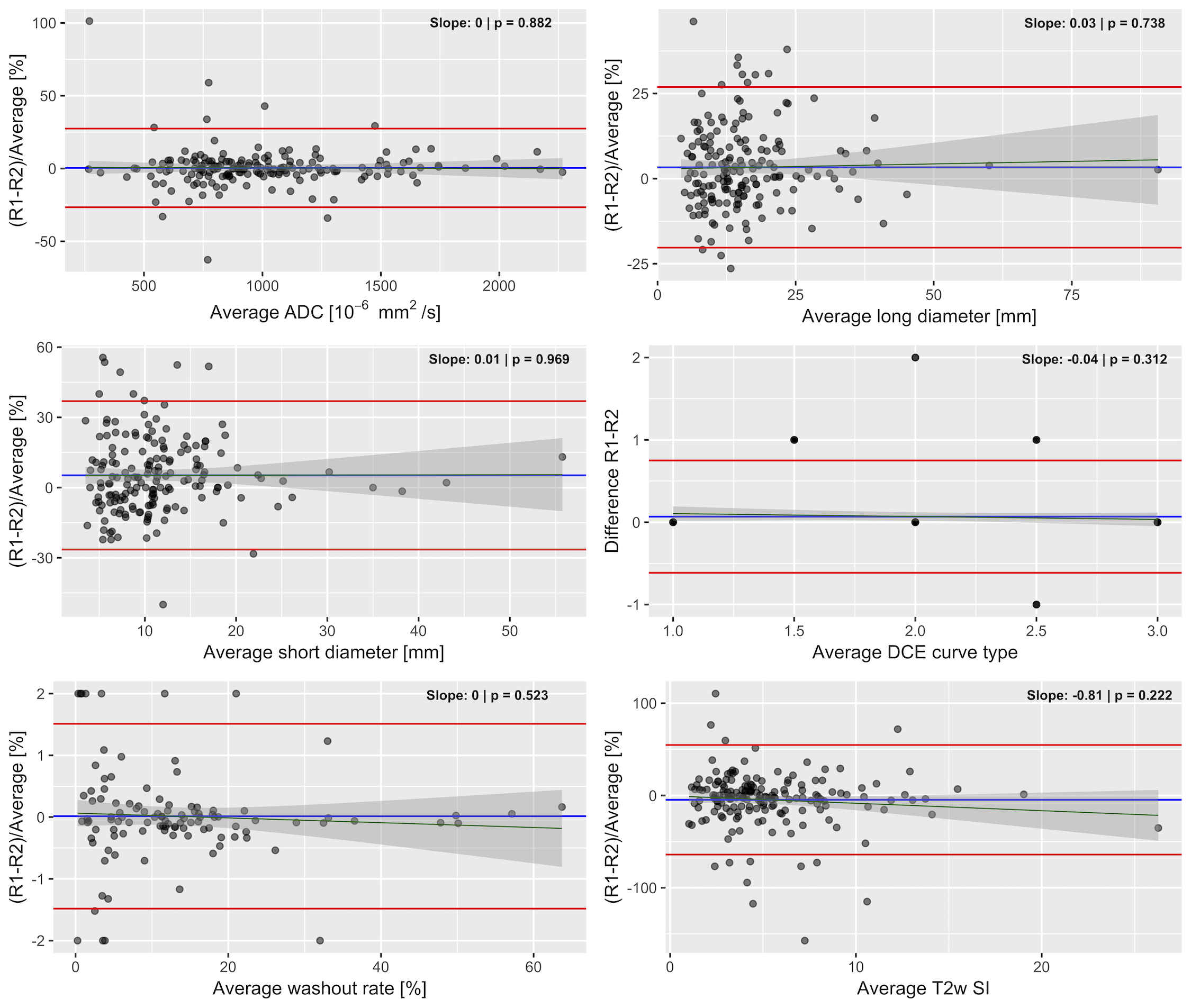

Supplement: S1 Fig — Systematic biases of the image parameter assessments between Reader 1 and 2. Bland-Altman plots depicting differences of the parameters against the average measurements, with mean difference (purple line) and 95% limits of agreement (red lines). The regression lines (in dark green) proved not to be significant for all parameters (-0,81 ≤ all slopes ≤ 0.03; all, p ≥ 0.222). (TIFF) [file pone.0228446.s001.tiff]

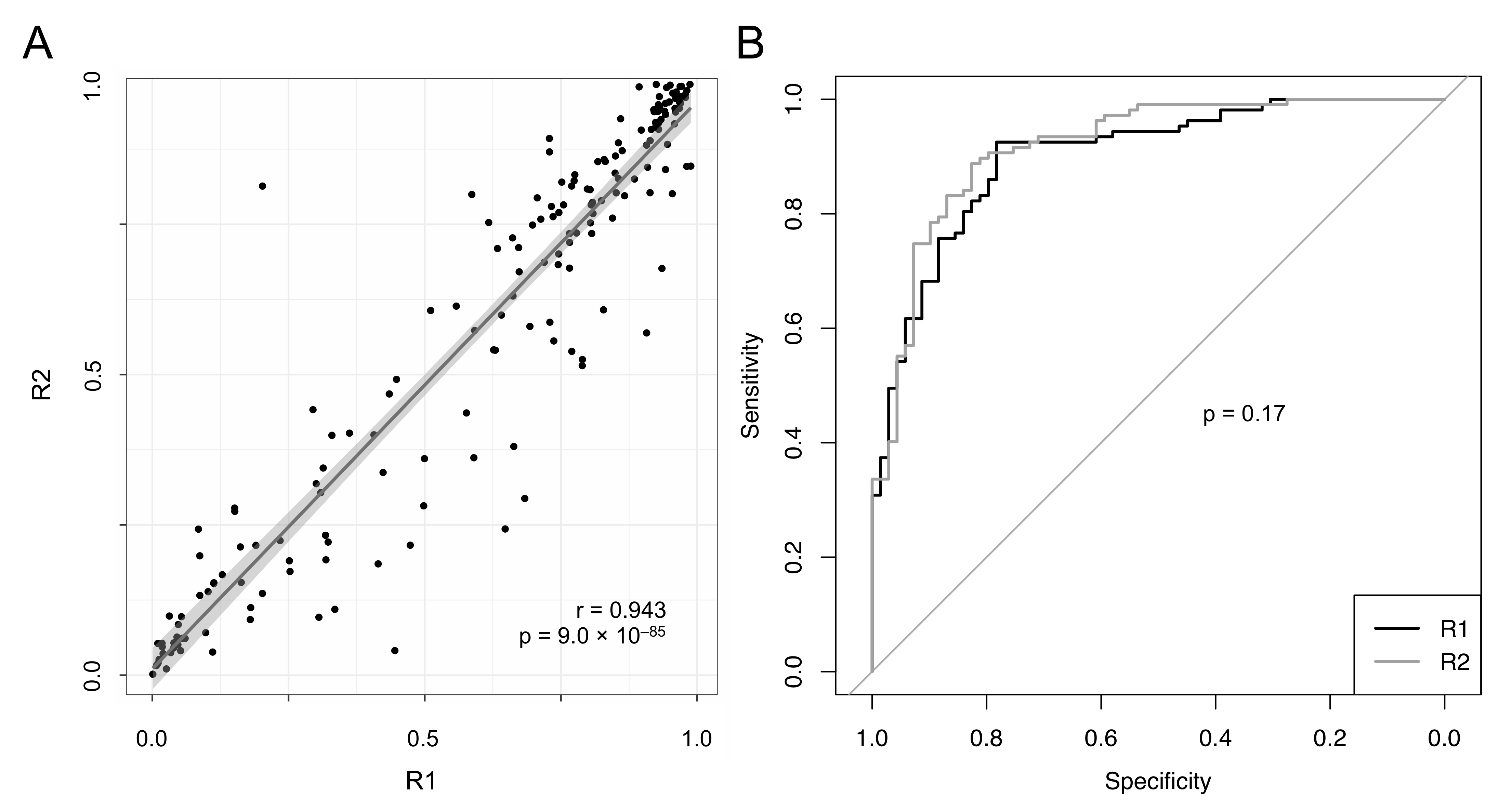

Supplement: S2 Fig — (A) Pearson plot depicting the correlation between the probability score outputs of the SVM algorithm for both readers (R1 and R2, respectively). Probability scores correlated strongly and highly significantly (r = 0.943; p = 9.0 × 10^−85^). (B) Receiver Operating Characteristic plots for R1 (black) and R2 (gray) with no significant difference (p = 0.17). (TIFF) [file pone.0228446.s002.tiff]
